# Circulating Hydrogen Sulfide (H_2_S) and Nitric Oxide (NO) Levels Are Significantly Reduced in HIV Patients Concomitant with Increased Oxidative Stress Biomarkers

**DOI:** 10.3390/jcm10194460

**Published:** 2021-09-28

**Authors:** Rahib K. Islam, Erinn Donnelly, Kazi N. Islam

**Affiliations:** 1 LSU Health Sciences Center, Department of Pharmacology, 1901 Perdido St., New Orleans, LA 70112, USA; rislam@xula.edu (R.K.I.); donn7171@vandals.uidaho.edu (E.D.); 2 Agricultural Research Development Program, College of Engineering, Science, Technology and Agriculture, Central State University, 1400 Brush Row Road, Wilberforce, OH 45384, USA

**Keywords:** H_2_S, HIV, cardiovascular disease, antioxidant, oxidative stress, Hs-CRP, IL-6

## Abstract

Human immunodeficiency virus (HIV) attacks the immune system and weakens the ability to fight infections/disease. Furthermore, HIV infection confers approximately two-fold higher risk of cardiac events compared with the general population. The pathological mechanisms responsible for the increased incidence of cardiovascular disease in HIV patients are largely unknown. We hypothesized that increased oxidative stress and attenuated circulating levels of the cardioprotective gaseous signaling molecules, nitric oxide (NO), and hydrogen sulfide (H_2_S) were involved in the cardiovascular pathobiology observed in HIV patients. Plasma samples from both HIV patients and age–matched normal subjects were used for all assays. Oxidative stress was determined by analyzing the levels of advanced oxidation protein products (AOPP) and H_2_O_2_. Antioxidant levels were determined by measuring the levels of trolox equivalent capacity. ADMA, hs-CRP, and IL-6 were determined by using ELISA. The levels of H_2_S (free H_2_S and sulfane sulfur) and NO_2_ (nitrite) were determined in the plasma samples by using gas chromatography and HPLC, respectively. In the present study we observed a marked induction in the levels of oxidative stress and decreased antioxidant status in the plasma of HIV patients as compared with the controls. Circulating levels of the cardiovascular disease biomarkers: ADMA, hs-CRP (high-sensitivity C-reactive protein), and IL-6 were significantly increased in the circulatory system of HIV patients. The levels of both nitrite and H_2_S/sulfane sulfur were significantly reduced in the plasma of HIV patients as compared with normal subjects. Our data demonstrate significant increases in circulating biomarkers of oxidative stress and cardiovascular (CV) in conjunction with decreased bioavailability of H_2_S and NO in HIV patients. Diminished levels of these two cardioprotective gaseous signaling molecules may be involved in the pathogenesis of CV disease in the setting of HIV.

## 1. Introduction

A gaseous signaling molecule, nitric oxide (NO) plays a pivotal role in cardiovascular homeostasis [1,2]. NO is synthesized endogenously via three isoforms of NO synthase (NOS) as well as by non-enzymatic reduction of nitrate (NO_3_^−^) and nitrite (NO_2_^−^). NO_3_^−^ and NO_2_^−^ are physiologically recycled in the blood and tissues, acting as precursors that can be easily converted to NO on demand [2]. The bioavailability of NO is reduced in cardiovascular disease states as primarily a result of increased oxidative stress and inflammation. Oxidative stress and inflammation result in increased breakdown of NO and reduced activity and dysregulated function of eNOS both of which result in significant reductions in NO and nitrite bioavailability [3,4,5].

Hydrogen sulfide (H_2_S) is a critical cell-signaling molecule required for cardiovascular homeostasis, much like NO [6,7,8]. The production of H_2_S in mammalian systems has been attributed to three endogenous enzymes: cystathionine β-synthase (CBS), cystathionine γ-lyase (CSE), and 3-mercaptopyruvate sulfur transferase (3-MST) [9]. Both endogenous and exogenous H_2_S respond to a wide range of protective actions including vasodilation, anti-inflammatory, antioxidant, anti-apoptotic, and modulation of cellular metabolism [10]. H_2_S is a powerful reducing agent and is likely to be consumed by endogenous oxidant species, such as peroxynitrite [11], superoxide [12], and hydrogen peroxide [13]. Although NO and H_2_S are thought to modulate independent pathways, there is evidence of crosstalk between these two gaseous signaling molecules [14,15]. Our group previously demonstrated that H_2_S plays a role in the protection against acute MI/R injury and HF [16]. Treatment with exogenous H_2_S or modulation of the endogenously produced H_2_S through the cardiac-specific overexpression of the H_2_S generating enzyme, CSE protects against acute MI/R injury and HF by attenuating oxidative stress, inhibiting apoptosis, and reducing inflammation [16,17]. Treatment with H_2_S therapy improves survival after cardiac arrest and cardiopulmonary resuscitation in an eNOS-dependent [18] manner and provides cardioprotection against MI/R injury by activating eNOS/NO. 

A number of studies have confirmed an association between markers of inflammation, as well as markers of NO regulation, and depressive symptoms in patients with heart failure (HF). Biomarkers indicating raised inflammatory activity, e.g., C-reactive protein (CRP) and interleukin 6 (IL-6), have been associated with an increased risk of future cardiovascular disease in healthy individuals, in patients with stable and unstable coronary artery disease, and in congestive heart failure patients [19,20]. Treated HIV is associated with chronic inflammatory changes, including chronic subclinical myocardial edema and a high incidence of pericardial effusions [21]. These chronic inflammatory changes may underlie the high incidence of myocardial fibrosis and alteration in cardiac function observed in patients with treated HIV [21]. HIV infection is associated with high rates of CVD complications, including acute myocardial infarction [22], sudden cardiac death [23], and HF [24]. People with HIV also have additional risk factors for cardiovascular disease (CVD) due to side effects of HIV medications. Evidence exists that certain antiretroviral medications and protease inhibitors used to treat these patients may increase the lifetime risk of developing CVD [25] in part because they can cause hyperlipidemia and insulin resistance. While the benefits of such medications clearly outweigh the risks, more study about what accounts for this effect is required. In order to better understand the overall cardiovascular health of these patients, plasma from HIV patients and healthy controls were tested for levels of asymmetric dimethylarginine (ADMA), a protein that has been linked to cardiovascular disease. Biomarkers of CVD such as high-sensitivity C-reactive protein (hs-CRP), and IL-6, a pro-inflammatory cytokine, were also determined. The status of cardioprotective gases, such as NO and H_2_S signaling in HIV plasma were also evaluated. Our study demonstrates that significant elevation in oxidative stress and biomarkers of CVD are associated with decreased bioavailability of H_2_S and NO in HIV patients. Decreased levels of these two gaseous signaling molecules may be involved in the pathogenesis of CVD in the setting of HIV.

## 2. Materials and Methods

Collection of normal and HIV plasma: Plasma samples from HIV patients were obtained from the LSUSHC-NO HIV Outpatient Clinic, New Orleans, LA, USA. All patients were consented to the use of their plasma for research purposes and signed a HIPPA release for use of the medical record. Institutional Review Board approval with consent was obtained. Age-matched single donor human plasma was purchased from Innovative Research.

### 2.1. Measurement of Advanced Oxidative Protein Products (AOPP)

All reagents or chemicals used in our experiments were purchased from Sigma-Aldrich, Milwaukee, WI, USA. Plasma advanced oxidation protein products (AOPP) as markers of protein oxidation were determined using an AOPP assay kit purchased from Abcam, Burlingame, CA, USA, catalogue, ab242295). Briefly, all reagents were equilibrated to room temperature prior to use. Either 200 μL of plasma samples (plasma sample was diluted in 1× assay diluent) or standards were added to separate wells of the microtiter plate and incubated with 10 μL of chloramine reaction initiator on a shaker for 5 min. Reaction was stopped by adding 20 μL of stop solution and the absorbance of each well was read immediately on a spectrophometric plate reader using 340 nm as the primary wavelength. The sample AOPP content was calculated by reference to the chloramine standard curve [26].

### 2.2. Measurement of Hydrogen Peroxide (H_2_O_2_)

H_2_O_2_ levels were measured in plasma obtained from both controls and HIV patients by using H_2_O_2_ assay kit purchased from Abcam, catalogue, ab102500. Plasma samples were centrifuged for 15 min at 1000× *g* and particulate pellet was removed. Either 50 μL plasma samples (plasma was diluted in assay buffer) or standards were added in each well of microtiter plate and 50 μL of reaction mixture was added to each test sample and H_2_O_2_ standards followed by incubation at room temperature for 10 min. Optical density was measured at 570 nm on a micro-plate reader. H_2_O_2_ concentration (mM) in each plasma sample was calculated from a H_2_O_2_ standard curve.

### 2.3. Measurement of Total Antioxidant Capacity

Total antioxidant capacity for plasma samples obtained from controls and HIV patients was measured by using Trolox equivalent antioxidant capacity assay kit from Abcam, catalogue, ab65329. Briefly, all reagents were equilibrated to room temperature prior to use. A Plasma sample of 0.1 μL without protein mask or 10 μL with protein mask was adjusted to 100 μL with ddH_2_O followed by addition of 100 μL of Cu^2+^ working solution to all standard and sample wells of microtiter plate. The plate was incubated at room temperature for 90 min on an orbital shaker protected from light. Optical density was measured at 570 nm on a micro-plate reader. 

### 2.4. Measurement of NO Metabolites

Plasma nitrite concentrations for both control and HIV subjects were quantified by ion high performance liquid chromatography HPLC (ENO20 Analyzer; Eicom, Kyoto, Japan).

### 2.5. ADMA Assay

The levels of ADMA in the plasma of controls and HIV patients were measured by using Enzyme-linked immunosorbent assay (ELISA) purchased from Immundiagnostik AG, Bensheim, Germany. catalogue, K7828. Briefly, first microtiter strips were washed with ELISA wash buffer followed by addition of 100 mL of derivatized standards or controls or plasma samples (plasma was diluted with reaction buffer) to respective well. 100 mL of diluted ADMA antibody (AB) was added into each well and incubated overnight at 2–8 °C. On the following day, the contents of each well were aspirated and washed with wash buffer and 200 mL of diluted peroxidase conjugate was added into each well. After 60 min of incubation at room temperature on a horizontal shaker (180–240 rpm), the contents of each well were aspirated, washed, and incubated with 200 mL of TMB substrate for 6–10 min at room temperature in the dark. Reaction was stopped by addition of 100 mL of stop solution and absorption was measured immediately at 450 nm against 620 nm as a reference.

### 2.6. Measurement of Hydrogen Sulfide (H_2_S) and Sulfane Sulfur

The levels of H_2_S and sulfane sulfur levels were determined in plasma samples of controls and HIV patients by using gas chromatography chemiluminescence according to the method published previously [27].

### 2.7. Measurement of High-Sensitivity C-Reactive Protein (hs-CRP)

The plasma levels of hs-CRP were determined both in controls and HIV plasma samples by utilizing an ELISA kit purchased from MyBioSource, Inc., San Diego, CA, USA, catalogue, MBS040244. All reagents were equilibrated to room temperature prior to use. Either 50 mL of standards or controls or plasma samples (plasma was diluted with sample diluent) were added to separate wells of microtiter strips. A total of 100 mL of HRP-conjugated reagent was added to each well and incubated for 60 min at 37 °C. After incubation, the contents of the well were aspirated and washed with wash buffer. A total of 50 mL of chromogen solution A and 50 mL of chromogen solution B were added to each well successively and incubated for 15 min at 37 °C in the dark. Reaction was stopped by adding 50 mL of stop solution and optical density was read at 450 nm using an ELISA reader within 15 min after adding stop solution.

### 2.8. Measurement of IL-6 

The levels of IL-6 were analyzed in plasma of controls and HIV patients by ELISA assay purchased from MyBioSource, Inc., San Diego, CA, USA, catalogue, MBS261259. The procedure in brief: 100 mL of standards or plasma samples (plasma was diluted with sample diluent) were added to separate wells (100 mL for each) of microtiter strips. The reaction wells were sealed with adhesive tapes, hatching in incubator at 37 °C for 90 min. After washing the plate, biotinylated human IL-6 antibody liquid was added to each well and sealed with adhesive tapes, hatching in incubator at 37 °C for 60 min. After washing enzyme-conjugate liquid was added to each well (100 mL for each) and reaction wells were sealed with adhesive tapes, hatching in incubator at 37 °C for 30 min. After washing with wash buffer 100 mL of color reagent liquid was added to individual wells, hatching in dark incubator at 37 °C. The chromogenic reaction was controlled within 30 min. A total of 100 mL of color reagent C was added to individual well and optical density was read at 450 nm within 10 min.

### 2.9. Statistical Analysis 

All data in this study were expressed as the mean ± SEM. Statistical significance between two groups was determined using the two-tailed Student’s *t* test. A *p* value of < 0.05 was considered statistically significant. The number inside the bar of each figure denotes the number of subjects per control or HIV group.

## 3. Results

**Figure 1:**
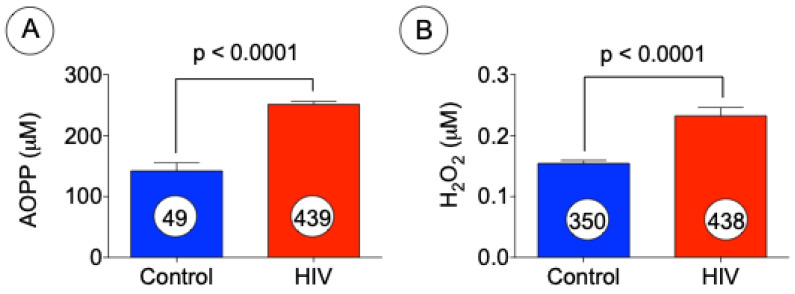
Oxidative stress in HIV patients. Levels of oxidative stress were determined by measuring the levels of AOPP (**A**) and H_2_O_2_ (**B**) in plasma samples obtained from control and HIV patients. The data presented above represent mean ± SE, *p* < 0.0001 versus control (*t* test).

First, we were interested to determine the levels of oxidative stress in patients infected with HIV. Markers of oxidative stress such as AOPP and H_2_O_2_ were assessed in plasma obtained from HIV patients and normal subjects. As shown in Figure 1A,B, the levels of both of these oxidative stress markers were significantly increased as compared with the control plasma. 

**Figure 2:**
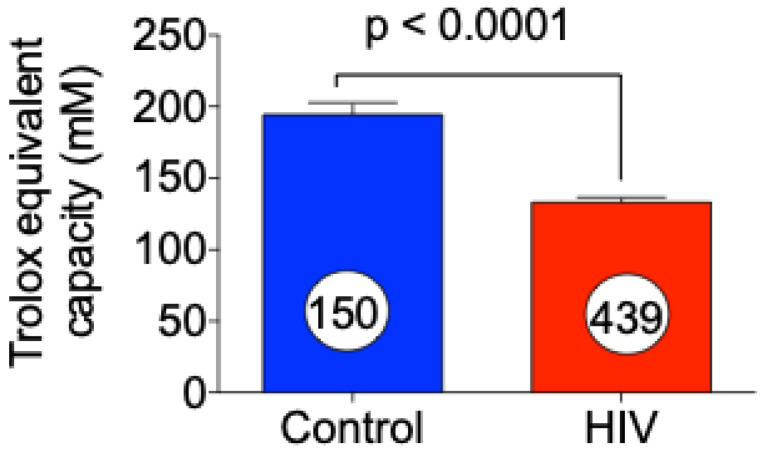
Reduction in total antioxidant capacity in HIV patients. Total antioxidant activity was determined by measuring the trolox equivalent capacity in the plasma of control and HIV patients. The data presented above represent mean ± SE, *p* < 0.0001 versus control (*t* test).

We then evaluated the levels of total antioxidant capacity by measuring Trolox equivalent capacity in plasma samples obtained from control and HIV patients. Trolox equivalent capacity or total antioxidant activity (activities of small molecules including protein molecules) was significantly reduced in HIV patients as compared with control plasma (Figure 2). The data in Figure 1; Figure 2 suggest that induction of oxidative stress in HIV patients is followed by reduction in the levels of antioxidant status in HIV patients.

**Figure 3:**
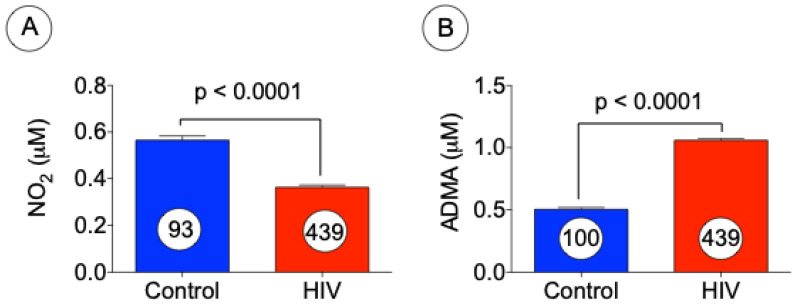
Levels of nitrite (NO_2_) and ADMA are inversely correlated in HIV patients. Levels of NO_2_ (**A**) were measured using HPLC and ADMA (**B**) was assessed by using ELISA in the plasma of normal subjects and HIV patients. The data presented above represent mean ± SE, *p* < 0.0001 versus control (*t* test).

To determine if HIV infection was associated with alterations in circulating NO bioavailability, we measured levels of the NO intermediate, nitrite, and an endogenous inhibitor of nitric oxide synthase (i.e., ADMA). We measured plasma nitrite levels using HPLC and ADMA using a commercial ELISA assay kit. Figure 3A,B shows the levels of nitrite were significantly reduced, and reciprocally ADMA was markedly increased in HIV patients as compared with control subjects. These findings indicate a significant reduction in NO bioavailability in HIV patients that may be a result of eNOS inhibition by the arginine analog ADMA.

**Figure 4:**
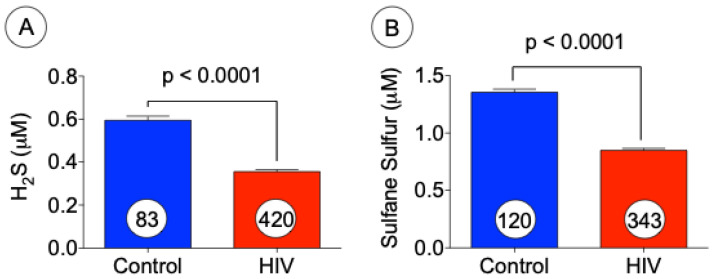
Decreased levels of H_2_S and sulfane sulfur in HIV patients. Levels of H2S (**A**) and sulfane sulfur (**B**) were measured using gas chromatography in the plasma samples obtained from normal subjects and HIV patients. The data presented above represent mean ± SE, *p* < 0.01 (**A**) and *p* < 0.0001 (**B**) versus control (*t* test).

We were interested to determine the levels of H_2_S in the plasma samples of both HIV and control subjects. We analyzed the levels of H_2_S and sulfane sulfur using gas chromatography coupled with sulfur chemiluminescence [27]. Marked reduction in the levels of both H_2_S and sulfane sulfur were observed in HIV patients as compared with controls (Figure 4A,B). The decreased levels of H_2_S/sulfane sulfur that we observed in plasma samples may be due to the elevation of oxidative stress in HIV patients. We found that the HIV population had significantly (*p* < 0.01) lower levels of hydrogen sulfide when compared with the controls. Sulfane sulfur (or thiosulfoxide) which is thought to be a biologically relevant storage pool of hydrogen sulfide [28] was similarly found to be significantly (*p* < 0.0001) decreased in patients with HIV as compared with control.

**Figure 5:**
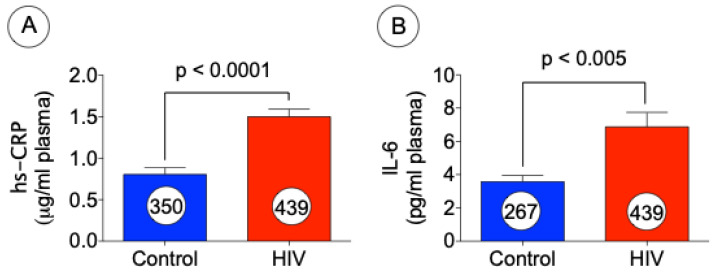
Increased hs-CRP and IL-6 levels in HIV patients. The levels of cardiovascular biomarkers, such as hs-CRP (**A**) and IL-6 (**B**) were measured by using ELISA assays. The data presented above represent mean ± SE, *p* < 0.0001 (**A**) and *p* < 0.005 (**B**) versus control (*t* test).

Both hs-CRP and IL-6 were found to be elevated in CVD. To correlate HIV with CVD we then measured the levels of hs-CRP and IL-6 in the plasma samples obtained from controls and HIV patients by utilizing ELISA assay. As can be seen both hs-CRP (Figure 5A) and IL-6 (Figure 5B) are significantly upregulated in HIV patients as compared with control subjects.

Our data demonstrate that significant induction in oxidative stress and biomarkers of cardiovascular disease are associated with decreased bioavailability of H_2_S and NO in HIV patients. Reduction in the levels of these two cardioprotective gaseous signaling molecules may be involved in the pathogenesis of cardiovascular disease in the setting of HIV.

## 4. Discussion

People with HIV are twice as likely as the general population to develop cardiovascular disease (CVD). A recent study [29] showed that CVD is a leading cause of death in people with HIV, accounting for approximately 11% of HIV patients deaths. While some risk factors for cardiovascular disease are common to both HIV and non-HIV populations, such as age, smoking, obesity and family history, people living with HIV have additional risk factors. The World Health Organization considers that HIV/AIDS and CVD will be in the top 3 causes for both global mortality and global disability-adjusted life-years in the year 2030 [30]. The hypothesis of the complex interplay of factors for cardiovascular disease is supported by the association of HIV with multiple vascular indices reflecting progressive stages of atherosclerosis, ranging from endothelial dysfunction [31] to coronary plaque itself [32]. Increased incidence of cardiovascular disease (CVD) occurs in HIV-infected patients compared with the general population [33]. There is increasing evidence that oxidative imbalance leads to increased cellular stress and results in alterations in molecular pathways that underpin the pathogenesis of several important human diseases, including heart disease, neurological disease, cancer, and ageing [34,35]. Antioxidant imbalance assessed by plasma malondialdehyde concentration and plasma total antioxidant ability is a condition which can contribute to increased destruction of CD4+ T cells and even disease progression if the balance is in favor of pro-oxidant (free radicals or reactive oxygen species (ROS) generation in HIV infected patients [36,37]. The mechanisms of coronary heart disease (CHD) among HIV-infected patients reflects a complex interplay of factors, including traditional risk factors, antiretroviral drug effects, and HIV-related parameters, such as inflammatory and immunologic changes [38,39].

The HIV virus causes chronic inflammation [40] due to constant activation of the immune system [41], which produces ROS. ROS are an important by-product of many cellular processes and are usually eliminated via interactions with superoxide dismutase, catalase, glutathione peroxidase, and peroxiredoxins. However, in many chronic conditions such as HIV, more ROS are produced than can be eliminated. This increased level of oxidants is toxic and leads to tissue damage. Other groups have recently documented the effect of oxidative stress in patients with HIV. A study by Mar Marsia et al. found that patients with HIV that died had significantly higher levels of F2-isoprostanes (F_2_IsoPs) and malondialdehyde (MDA) in their plasma than their matched controls [41]. One of the milestone findings in the redox biology of during antiretroviral therapy (ART) HIV-1 was the induction of oxidative stress. Numerous reports show that nucleoside and nonnucleoside reverse transcriptase (RT) inhibitors, as well as inhibitors of the viral protease, trigger massive ROS production in various cell types [42,43,44,45,46,47,48]. Series of studies reported an increase in oxidative stress additional to the persistent redox imbalance associated with HIV-1 infection manifested by an increase in oxidants and a decrease in antioxidant serum levels [49,50]. It is generally acknowledged that the components of ART may contribute to the development of cardiovascular diseases and CNS pathologies. The exact impact of oxidative stress on the efficacy of ART and HIV-1/AIDS progression and the molecular mechanisms of the redox imbalance in ART-treated HIV-infected individuals are still obscure and require further comprehensive studies. Although ART is able to clear viremia and improve the immunological condition of HIV-infected individuals for prolonged time, the virus rebounds to levels comparable to those observed before treatment initiation shortly after treatment withdrawal due to intactness of the major cellular reservoirs for HIV; central and transitional memory T-cells which harbor the transcriptionally silent form of viral DNA are not affected by classical antiretroviral drug regimens. 

Our findings support the existing literature about cardiovascular disease in HIV patients, much of which centers around the roles of oxidative stress and inflammation plays in the progression of the disease. Advanced oxidative protein products (AOPP) formed when the plasma proteins in the blood are oxidized by ROS and are indicative of oxidative stress. [51] Hydrogen peroxide (H_2_O_2_) is another known marker of oxidative stress. H_2_O_2_ acts as a signaling molecule [52] for the immune system and helps to recruit white blood cells to initiate healing to damaged tissues [53]. However, H_2_O_2_ easily breaks down into a hydroxyl radical, which can cause cellular damage. In conditions with chronic immune system activation and inflammation, H_2_O_2_ levels tend to be higher. We were interested to investigate the levels of oxidative status in the plasma of HIV patients. We observed the elevated levels of AOPP and H_2_O_2_ and decreased levels of antioxidant activity in HIV patients as compared with the control subjects. Antioxidants are among the body’s first line of defense against ROS/oxidative stress. The link between reduced antioxidant capacity, chronic inflammation and CVD has been well established. For example, a recent study found that reduced levels of antioxidants, along with chronic inflammation, are thought to be at least partially responsible for the increased risk of CVD for patients with rheumatoid arthritis (RA) [54]. Additionally, another study found a strong correlation between total antioxidant capacity (TAC) and the HIV disease progression, with healthy controls having the highest levels of antioxidants, symptomatic HIV patients having the lowest, and asymptomatic HIV patients’ levels in between the two [55]. We similarly found that the HIV population had significantly lower levels of antioxidant capacity when compared with the control population. It can be noted that antioxidant molecules may play protective roles against HIV infection and therefore, it will be reasonable to investigate the in vivo mechanism of actions of antioxidant molecules. It is well known that antioxidants are protective agents against cardiovascular, degenerative pathologies, and cancer, the actual contribution of these compounds to the maintenance of health and their in vivo mechanism of action are not yet known [56]. Previously published data have shown that a strong reverse correlation between the intake of fruits and vegetables and the occurrence of degenerative diseases and cancer [56]. The observed effect may be due to the synergistic action between the compounds of the endogenous antioxidant system (such as superoxide dismutase, glutathione peroxidase, glutathione -S-transferase, and catalase) and the antioxidants from the diet [56]. It can be noted here that polyphenols are beneficial plant compound with antioxidant properties that may help keep healthy and protect against various diseases. Polyphenols can be subdivided into flavonoids, phenolic acid, polyphenolic amides, and other polyphenols.

IL-6 is known as a biomarker of systemic inflammation and is elevated in cardiovascular diseases (CVD) and is also associated with oxidative stress. In addition, IL-6 has recently been implicated as a predictor of clinical events, serious non-AIDS conditions (SNA) and death in HIV patients [57,58]. Elevated levels of IL-6 were also found to be associated with an increased risk of atherosclerosis, even when other risk factors for CVD are controlled for [59]. IL-6 plays an important role in atherogenesis and pathogenesis of CVD. It can stimulate hepatic synthesis of acute-phase proteins, activate endothelial cells, promote lymphocyte proliferation, neutrophil migration, and macrophage differentiation [60,61]. In the general population, higher plasma IL-6 levels have been associated with greater carotid IMT [62], atherosclerosis progression [62], coronary heart disease [63] and CV deaths [64]. Similarly, in HIV-infected individuals on ART, higher plasma IL-6 levels have been associated with CVD [65], CV deaths [66], and mortality [67]. There is also evidence to suggest that IL-6 is a stronger predictor of clinical events than hsCRP or D-Dimer in HIV infection [57]. A vascular inflammation marker, hs-CRP is also increased during inflammatory disease states. Persistent high levels of CRP have been well correlated to risk of arteriosclerosis and CVD. A recent study found that overweight and obese patients with HIV had higher levels of CRP before and after beginning antiretroviral therapy (ART) when compared with underweight patients. Additionally, it was reported that although both groups gained weight after the initiation of ART, the underweight population showed proportional reduction in CRP levels for each BMI unit gained, while the overweight population showed an increase [68]. Another recent study found a strong correlation between high baseline hs-CRP levels in untreated HIV patients and early mortality after beginning ART [69]. In our study the changes in biomarkers of CVD, such as IL-6 and hs-CRP, were tested in both control subjects and HIV patients, and we observed marked induction of hs-CRP and IL-6 levels in patients with HIV when compared with uninfected controls. Based on this observation our conclusion is that HIV patients have a high risk for developing CVD. 

NO is a powerful gaseous signaling molecule plays an important role in maintaining the normal physiology of the heart and circulation. NO promotes vasodilation and regulates homeostasis between the antioxidant and oxidant systems. NO reacts with oxygen very rapidly to form nitrite (NO_2_) in the blood stream or in tissue and is readily converted back into NO under hypoxia or other stressful conditions to protect the cells against damage. ADMA is an endogenous inhibitor of nitric oxide synthases (NOS) that limits NO bioavailability and can increase production of NOS derived reactive oxidative species [70]. Increased plasma ADMA is one of the strongest predictors of mortality in patients who have had a myocardial infarction or suffer from chronic left heart failure, and is also an independent risk factor for several other conditions that contribute to heart failure development, including hypertension, coronary artery disease/atherosclerosis, diabetes, and renal dysfunction [70]. It has been reported that subjects with HIV suffer from reduced left ventricular (LV) ejection fraction and increased mass, which may be related to the increased cardiac morbidity and mortality in the population with HIV infection [21]. The changes in LV mass were independent of hypertension and body mass index [21]. In HIV-infected patients, while little is known about ADMA, studies have found increased levels in HIV-infected patients compared to uninfected controls [71,72]. Levels of ADMA also decrease with ART initiation [73]. Other studies have showed that elevated levels of ADMA are associated with higher viral loads, lower CD4+ counts, and high risk of pulmonary artery hypertension in HIV patients [74,75]. In the present study we found that NO bioavailability was attenuated concomitant with increased circulating levels of ADMA. It is unclear if the reduced NO levels are a result of increased ADMA resulting in nitric oxide synthase inhibition. 

There is a paucity of data on the role of H_2_S on HIV. H_2_S is another important endogenously produced gas that exerts important physiological actions. H_2_S also promotes the production of NO and relaxation of vascular smooth muscle [76]. H_2_S acts on eNOS to increase enzymatic production of NO following phosphorylation of ser1177 on eNOS [27]. H_2_S exerts very potent antioxidant properties and is cardioprotective. Deficiency of both NO and H_2_S is commonly observed in CVD [14,77]. Levels of H_2_S have also been shown to be decreased during development of hypertension in diabetic rats [78]. We were curious to investigate the status of H_2_S in HIV plasma samples. We observed that decreased levels of H_2_S are accompanied with decreased levels of NO in HIV patients as compared with control subjects. Antiviral and anti-inflammatory effects of H2S highlight its potential as a therapeutic molecule. It can, therefore, bolster the efficacy of the regular drug regime used for viral infections. Despite all these observations there is a dearth of knowledge in terms of molecular mechanism of these effects which could form a promising line of research. 

Our data is consistent with many of the recent studies on HIV and CVD. We found that HIV patients exhibit significantly elevated circulating levels of CVD risk biomarkers coupled with CVD and decreased antioxidant protection. More research is needed to determine the specific pathways that result in increased susceptibility of HIV patients to CVD. Further studies could allow for the development of novel therapeutics that can target these pathways.

## Data Availability

The data presented in this study are available on request from the corresponding author in accordance with the state regulations and appropriate laws.

## References

[1] Lundberg J.O., Weitzberg E., Gladwin M.T. (2008). The nitrate-nitrite-nitric oxide pathway in physiology and therapeutics. Nat. Rev. Drug Discov..

[2] Torregrossa A.C., Aranke M., Bryan N.S. (2011). Nitric oxide and geriatrics: Implications in diagnostics and treatment of the elderly. J. Geriatr. Cardiol. JGC.

[3] Forstermann U., Li H. (2011). Therapeutic effect of enhancing endothelial nitric oxide synthase (eNOS) expression and preventing eNOS uncoupling. Br. J. Pharmacol..

[4] Forstermann U., Sessa W.C. (2012). Nitric oxide synthases: Regulation and function. Eur. Heart J..

[5] Gomaraschi M., Ossoli A., Favari E., Adorni M.P., Sinagra G., Cattin L., Veglia F., Bernini F., Franceschini G., Calabresi L. (2013). Inflammation impairs eNOS activation by HDL in patients with acute coronary syndrome. Cardiovasc. Res..

[6] Li L., Rose P., Moore P.K. (2011). Hydrogen sulfide and cell signaling. Annu. Rev. Pharmacol. Toxicol..

[7] Lowicka E., Beltowski J. (2007). Hydrogen sulfide (H2S)-the third gas of interest for pharmacologists. Pharmacol. Rep..

[8] Szabo C. (2007). Hydrogen sulphide and its therapeutic potential. Nat. Rev. Drug Discov..

[9] Kimura H. (2011). Hydrogen sulfide: Its production, release and functions. Amino Acids.

[10] Wang R. (2002). Two’s company, three’s a crowd: Can H2S be the third endogenous gaseous transmitter?. FASEB J..

[11] Whiteman M., Armstrong J.S., Chu S.H., Jia-Ling S., Wong B.S., Cheung N.S., Halliwell B., Moore P.K. (2004). The novel neuromodulator hydrogen sulfide: An endogenous peroxynitrite ‘scavenger’?. J. Neurochem..

[12] Chang L., Geng B., Yu F., Zhao J., Jiang H., Du J., Tang C. (2008). Hydrogen sulfide inhibits myocardial injury induced by homocysteine in rats. Amino Acids.

[13] Geng B., Yang J., Qi Y., Zhao J., Pang Y., Du J., Tang C. (2004). H2S generated by heart in rat and its effects on cardiac function. Biochem. Biophys. Res. Commun..

[14] Kondo K., Bhushan S., King A.L., Prabhu S.D., Hamid T., Koenig S., Murohara T., Predmore B.L., Gojon G., Gojon G. (2013). H(2)S protects against pressure overload-induced heart failure via upregulation of endothelial nitric oxide synthase. Circulation.

[15] Predmore B.L., Lefer D.J., Gojon G. (2012). Hydrogen sulfide in biochemistry and medicine. Antioxid. Redox Signal..

[16] Calvert J.W., Jha S., Gundewar S., Elrod J.W., Ramachandran A., Pattillo C.B., Kevil C.G., Lefer D.J. (2009). Hydrogen sulfide mediates cardioprotection through Nrf2 signaling. Circ. Res..

[17] Elrod J.W., Calvert J.W., Morrison J., Doeller J.E., Kraus D.W., Tao L., Jiao X., Scalia R., Kiss L., Szabo C. (2007). Hydrogen sulfide attenuates myocardial ischemia-reperfusion injury by preservation of mitochondrial function. Proc. Natl. Acad. Sci. USA.

[18] Minamishima S., Bougaki M., Sips P.Y., Yu J.D., Minamishima Y.A., Elrod J.W., Lefer D.J., Bloch K.D., Ichinose F. (2009). Hydrogen sulfide improves survival after cardiac arrest and cardiopulmonary resuscitation via a nitric oxide synthase 3-dependent mechanism in mice. Circulation.

[19] Ridker P.M., Cannon C.P., Morrow D., Rifai N., Rose L.M., McCabe C.H., Pfeffer M.A., Braunwald E. (2005). Pravastatin or Atorvastatin E and Infection Therapy-Thrombolysis in Myocardial Infarction I. C-reactive protein levels and outcomes after statin therapy. N. Engl. J. Med..

[20] Yin W.H., Chen J.W., Jen H.L., Chiang M.C., Huang W.P., Feng A.N., Young M.S., Lin S.J. (2004). Independent prognostic value of elevated high-sensitivity C-reactive protein in chronic heart failure. Am. Heart J..

[21] Ntusi N., O’Dwyer E., Dorrell L., Wainwright E., Piechnik S., Clutton G., Hancock G., Ferreira V., Cox P., Badri M. (2016). HIV-1-Related Cardiovascular Disease Is Associated with Chronic Inflammation, Frequent Pericardial Effusions, and Probable Myocardial Edema. Circ. Cardiovasc. Imaging.

[22] Freiberg M.S., Chang C.C., Kuller L.H., Skanderson M., Lowy E., Kraemer K.L., Butt A.A., Bidwell Goetz M., Leaf D., Oursler K.A. (2013). HIV infection and the risk of acute myocardial infarction. JAMA Intern. Med..

[23] Tseng Z.H., Secemsky E.A., Dowdy D., Vittinghoff E., Moyers B., Wong J.K., Havlir D.V., Hsue P.Y. (2012). Sudden cardiac death in patients with human immunodeficiency virus infection. J. Am. Coll. Cardiol..

[24] Butt A.A., Chang C.C., Kuller L., Goetz M.B., Leaf D., Rimland D., Gibert C.L., Oursler K.K., Rodriguez-Barradas M.C., Lim J. (2011). Risk of heart failure with human immunodeficiency virus in the absence of prior diagnosis of coronary heart disease. Arch. Intern. Med..

[25] Kingery J.R., Alfred Y., Smart L.R., Nash E., Todd J., Naguib M.R., Downs J.A., Kalluvya S., Kataraihya J.B., Peck R.N. (2016). Short-term and long-term cardiovascular risk, metabolic syndrome and HIV in Tanzania. Heart.

[26] Granger D.A., Cicchetti D., Rogosch F.A., Hibel L.C., Teisl M., Flores E. (2007). Blood contamination in children’s saliva: Prevalence, stability, and impact on the measurement of salivary cortisol, testosterone, and dehydroepiandrosterone. Psychoneuroendocrinology.

[27] King A.L., Polhemus D.J., Bhushan S., Otsuka H., Kondo K., Nicholson C.K., Bradley J.M., Islam K.N., Calvert J.W., Tao Y.X. (2014). Hydrogen sulfide cytoprotective signaling is endothelial nitric oxide synthase-nitric oxide dependent. Proc. Natl. Acad. Sci. USA.

[28] Toohey J.I., Cooper A.J. (2014). Thiosulfoxide (sulfane) sulfur: New chemistry and new regulatory roles in biology. Molecules.

[29] Smith C.J., Ryom L., Weber R., Morlat P., Pradier C., Reiss P., Kowalska J.D., de Wit S., Law M., el Sadr W. (2014). Trends in underlying causes of death in people with HIV from 1999 to 2011 (D:A:D): A multicohort collaboration. Lancet.

[30] Mathers C.D., Loncar D. (2006). Projections of global mortality and burden of disease from 2002 to 2030. PLoS Med..

[31] Francisci D., Giannini S., Baldelli F., Leone M., Belfiori B., Guglielmini G., Malincarne L., Gresele P. (2009). HIV type 1 infection, and not short-term HAART, induces endothelial dysfunction. Aids.

[32] Lo J., Abbara S., Shturman L., Soni A., Wei J., Rocha-Filho J.A., Nasir K., Grinspoon S.K. (2010). Increased prevalence of subclinical coronary atherosclerosis detected by coronary computed tomography angiography in HIV-infected men. Aids.

[33] Hakeem A., Bhatti S., Cilingiroglu M. (2010). The spectrum of atherosclerotic coronary artery disease in HIV patients. Curr. Atheroscler. Rep..

[34] Pacher P., Szabo C. (2008). Role of the peroxynitrite-poly(ADP-ribose) polymerase pathway in human disease. Am. J. Pathol..

[35] Vassalle C., Pratali L., Boni C., Mercuri A., Ndreu R. (2008). An oxidative stress score as a combined measure of the pro-oxidant and anti-oxidant counterparts in patients with coronary artery disease. Clin. Biochem..

[36] Fulbert J.C., Cals M.J. (1992). Free radicals in clinical biology. Origin, pathogenic effect and defense mechanisms. Pathol.-Biol..

[37] Halliwell B., Cross C.E. (1991). Reactive oxygen species, antioxidants, and acquired immunodeficiency syndrome. Sense or speculation?. Arch. Intern. Med..

[38] Lang S., Mary-Krause M., Cotte L., Gilquin J., Partisani M., Simon A., Boccara F., Costagliola D., Clinical Epidemiology Group of the French Hospital Database on HIV (2010). Impact of individual antiretroviral drugs on the risk of myocardial infarction in human immunodeficiency virus-infected patients: A case-control study nested within the French Hospital Database on HIV ANRS cohort CO4. Arch. Intern. Med..

[39] Worm S.W., Sabin C., Weber R., Reiss P., El-Sadr W., Dabis F., De Wit S., Law M., Monforte A.D., Friis-Moller N. (2010). Risk of myocardial infarction in patients with HIV infection exposed to specific individual antiretroviral drugs from the 3 major drug classes: The data collection on adverse events of anti-HIV drugs (D:A:D) study. J. Infect. Dis..

[40] Appay V., Sauce D. (2008). Immune activation and inflammation in HIV-1 infection: Causes and consequences. J. Pathol..

[41] Masia M., Padilla S., Fernandez M., Rodriguez C., Moreno A., Oteo J.A., Antela A., Moreno S., Del Amo J., Gutierrez F. (2016). Oxidative Stress Predicts All-Cause Mortality in HIV-Infected Patients. PLoS ONE.

[42] Wang X., Chai H., Lin P.H., Yao Q., Chen C. (2009). Roles and mechanisms of human immunodeficiency virus protease inhibitor ritonavir and other anti-human immunodeficiency virus drugs in endothelial dysfunction of porcine pulmonary arteries and human pulmonary artery endothelial cells. Am. J. Pathol..

[43] Nagiah S., Phulukdaree A., Chuturgoon A. (2015). Mitochondrial and Oxidative Stress Response in HepG2 Cells Following Acute and Prolonged Exposure to Antiretroviral Drugs. J. Cell Biochem..

[44] Manda K.R., Banerjee A., Banks W.A., Ercal N. (2011). Highly active antiretroviral therapy drug combination induces oxidative stress and mitochondrial dysfunction in immortalized human blood-brain barrier endothelial cells. Free Radic. Biol. Med..

[45] Mondal D., Pradhan L., Ali M., Agrawal K.C. (2004). HAART drugs induce oxidative stress in human endothelial cells and increase endothelial recruitment of mononuclear cells: Exacerbation by inflammatory cytokines and amelioration by antioxidants. Cardiovasc. Toxicol..

[46] Hurwitz B.E., Klimas N.G., Llabre M.M., Maher K.J., Skyler J.S., Bilsker M.S., McPherson-Baker S., Lawrence P.J., Laperriere A.R., Greeson J.M. (2004). HIV, metabolic syndrome X, inflammation, oxidative stress, and coronary heart disease risk: Role of protease inhibitor exposure. Cardiovasc. Toxicol..

[47] Chandra S., Mondal D., Agrawal K.C. (2009). HIV-1 protease inhibitor induced oxidative stress suppresses glucose stimulated insulin release: Protection with thymoquinone. Exp. Biol. Med..

[48] Weiss M., Kost B., Renner-Muller I., Wolf E., Mylonas I., Bruning A. (2016). Efavirenz Causes Oxidative Stress, Endoplasmic Reticulum Stress, and Autophagy in Endothelial Cells. Cardiovasc. Toxicol..

[49] Masia M., Padilla S., Bernal E., Almenar M.V., Molina J., Hernandez I., Graells M.L., Gutierrez F. (2007). Influence of antiretroviral therapy on oxidative stress and cardiovascular risk: A prospective cross-sectional study in HIV-infected patients. Clin. Ther..

[50] Mandas A., Iorio E.L., Congiu M.G., Balestrieri C., Mereu A., Cau D., Dessi S., Curreli N. (2009). Oxidative imbalance in HIV-1 infected patients treated with antiretroviral therapy. J. Biomed. Biotechnol..

[51] Marsche G., Frank S., Hrzenjak A., Holzer M., Dirnberger S., Wadsack C., Scharnagl H., Stojakovic T., Heinemann A., Oettl K. (2009). Plasma-advanced oxidation protein products are potent high-density lipoprotein receptor antagonists in vivo. Circ. Res..

[52] Veal E.A., Day A.M., Morgan B.A. (2007). Hydrogen peroxide sensing and signaling. Mol. Cell.

[53] Niethammer P., Grabher C., Look A.T., Mitchison T.J. (2009). A tissue-scale gradient of hydrogen peroxide mediates rapid wound detection in zebrafish. Nature.

[54] Bindal U.D., Saxena R., Siddiqui M.H., Sharma D. (2016). Correlation of Paraoxonase Status with Disease Activity Score and Systemic Inflammation in Rheumatoid Arthritic Patients. J. Clin. Diagn. Res. JCDR.

[55] Suresh D.R., Annam V., Pratibha K., Prasad B.V. (2009). Total antioxidant capacity—A novel early bio-chemical marker of oxidative stress in HIV infected individuals. J. Biomed. Sci..

[56] Metere A., Giacomelli L. (2017). Absorption, metabolism and protective role of fruits and vegetables polyphenols against gastric cancer. Eur. Rev. Med. Pharmacol. Sci..

[57] Borges A.H., O’Connor J.L., Phillips A.N., Neaton J.D., Grund B., Neuhaus J., Vjecha M.J., Calmy A., Koelsch K.K., Lundgren J.D. (2016). Interleukin 6 Is a Stronger Predictor of Clinical Events Than High-Sensitivity C-Reactive Protein or D-Dimer During HIV Infection. J. Infect. Dis..

[58] Grund B., Baker J.V., Deeks S.G., Wolfson J., Wentworth D., Cozzi-Lepri A., Cohen C.J., Phillips A., Lundgren J.D., Neaton J.D. (2016). Relevance of Interleukin-6 and D-Dimer for Serious Non-AIDS Morbidity and Death among HIV-Positive Adults on Suppressive Antiretroviral Therapy. PLoS ONE.

[59] Hsu D.C., Ma Y.F., Hur S., Li D., Rupert A., Scherzer R., Kalapus S.C., Deeks S., Sereti I., Hsue P.Y. (2016). Plasma IL-6 levels are independently associated with atherosclerosis and mortality in HIV-infected individuals on suppressive ART. Aids.

[60] Hartman J., Frishman W.H. (2014). Inflammation and atherosclerosis: A review of the role of interleukin-6 in the development of atherosclerosis and the potential for targeted drug therapy. Cardiol. Rev..

[61] Hunter C.A., Jones S.A. (2015). IL-6 as a keystone cytokine in health and disease. Nat. Immunol..

[62] Thakore A.H., Guo C.Y., Larson M.G., Corey D., Wang T.J., Vasan R.S., D’Agostino R.B., Sr Lipinska I., Keaney J.F., Benjamin E.J. (2007). Association of multiple inflammatory markers with carotid intimal medial thickness and stenosis (from the Framingham Heart Study). Am. J. Cardiol..

[63] Danesh J., Kaptoge S., Mann A.G., Sarwar N., Wood A., Angleman S.B., Wensley F., Higgins J.P., Lennon L., Eiriksdottir G. (2008). Long-term interleukin-6 levels and subsequent risk of coronary heart disease: Two new prospective studies and a systematic review. PLoS Med..

[64] Harris T.B., Ferrucci L., Tracy R.P., Corti M.C., Wacholder S., Ettinger W.H., Heimovitz H., Cohen H.J., Wallace R. (1999). Associations of elevated interleukin-6 and C-reactive protein levels with mortality in the elderly. Am. J. Med..

[65] Duprez D.A., Neuhaus J., Kuller L.H., Tracy R., Belloso W., De Wit S., Drummond F., Lane H.C., Ledergerber B., Lundgren J. (2012). Inflammation, coagulation and cardiovascular disease in HIV-infected individuals. PLoS ONE.

[66] Nordell A.D., McKenna M., Borges A.H., Duprez D., Neuhaus J., Neaton J.D., Insight Smart E.S.G., Committee S.S. (2014). Severity of cardiovascular disease outcomes among patients with HIV is related to markers of inflammation and coagulation. J. Am. Heart Assoc..

[67] Kuller L.H., Tracy R., Belloso W., De Wit S., Drummond F., Lane H.C., Ledergerber B., Lundgren J., Neuhaus J., Nixon D. (2008). Inflammatory and coagulation biomarkers and mortality in patients with HIV infection. PLoS Med..

[68] Mave V., Erlandson K.M., Gupte N., Balgopal A., Asmuth D.M., Campbell T.B., Smeaton L., Kumarasamy N., Hakim J., Santos B. (2016). Inflammation and Change in Body Weight with Antiretroviral Therapy Initiation in a Multinational Cohort of HIV-infected Adults. J. Infect. Dis..

[69] Ledwaba L., Tavel J.A., Khabo P., Maja P., Qin J., Sangweni P., Liu X., Follmann D., Metcalf J.A., Orsega S. (2012). Pre-ART levels of inflammation and coagulation markers are strong predictors of death in a South African cohort with advanced HIV disease. PLoS ONE.

[70] Liu X., Hou L., Xu D., Chen A., Yang L., Zhuang Y., Xu Y., Fassett J.T., Chen Y. (2016). Effect of asymmetric dimethylarginine (ADMA) on heart failure development. Nitric Oxide.

[71] Kurz K., Teerlink T., Sarcletti M., Weiss G., Zangerle R., Fuchs D. (2009). Plasma concentrations of the cardiovascular risk factor asymmetric dimethylarginine (ADMA) are increased in patients with HIV-1 infection and correlate with immune activation markers. Pharmacol. Res..

[72] Haissman J.M., Haugaard A.K., Knudsen A., Kristoffersen U.S., Seljeflot I., Pedersen K.K., Lebech A.M., Hasbak P., Kjaer A., Ostrowski S.R. (2016). Marker of Endothelial Dysfunction Asymmetric Dimethylarginine Is Elevated in HIV Infection but Not Associated with Subclinical Atherosclerosis. J. Acquir. Immune Defic. Syndr..

[73] Baker J.V., Neuhaus J., Duprez D., Freiberg M., Bernardino J.I., Badley A.D., Nixon D.E., Lundgren J.D., Tracy R.P., Neaton J.D. (2012). HIV replication, inflammation, and the effect of starting antiretroviral therapy on plasma asymmetric dimethylarginine, a novel marker of endothelial dysfunction. J. Acquir. Immune Defic. Syndr..

[74] Parikh R.V., Scherzer R., Grunfeld C., Nitta E.M., Leone A., Martin J.N., Deeks S.G., Ganz P., Hsue P.Y. (2013). Elevated levels of asymmetric dimethylarginine are associated with lower CD4+ count and higher viral load in HIV-infected individuals. Atherosclerosis.

[75] Parikh R.V., Scherzer R., Nitta E.M., Leone A., Hur S., Mistry V., Macgregor J.S., Martin J.N., Deeks S.G., Ganz P. (2014). Increased levels of asymmetric dimethylarginine are associated with pulmonary arterial hypertension in HIV infection. Aids.

[76] Wang R. (2003). The Gasotransmitter Role oif Hydrogen Sulfide. Antioxid. Redox Signal..

[77] Polhemus D.J., Kondo K., Bhushan S., Bir S.C., Kevil C.G., Murohara T., Lefer D.J., Calvert J.W. (2013). Hydrogen sulfide attenuates cardiac dysfunction after heart failure via induction of angiogenesis. Circ. Heart Fail..

[78] El-Sayed S.S., Zakaria M.N., Abdel-Ghany R.H., Abdel-Rahman A.A. (2016). Cystathionine-gamma lyase-derived hydrogen sulfide mediates the cardiovascular protective effects of moxonidine in diabetic rats. Eur. J. Pharmacol..

